# Factors associated with the failure of obstetric fistula repair in Guinea: implications for practice

**DOI:** 10.1186/s12978-016-0248-3

**Published:** 2016-11-08

**Authors:** Alexandre Delamou, Therese Delvaux, Abdoul Habib Beavogui, Abdoulaye Toure, Delphin Kolié, Sidikiba Sidibé, Mandian Camara, Kindy Diallo, Thierno Hamidou Barry, Moustapha Diallo, Alain Leveque, Wei-Hong Zhang, Vincent De Brouwere

**Affiliations:** 1 Ecole de Santé Publique, Université libre de Bruxelles (ULB), Bruxelles, Belgium; 2 Centre national de Formation et de Recherche en Santé Rurale de Maferinyah, Forécariah, Guinea; 3 Department of Public Health, Institute of Tropical Medicine, Antwerp, Belgium; 4 Department of Public Health, Faculty of Medicine, University of Conakry, Conakry, Guinea; 5 Centre Médicosocial Jean Paul II de Conakry, Conakry, Guinea; 6 Hôpital Régional de Labé, Labé, Guinea; 7 Hôpital préfectoral de Kissidougou, Kissidougou, Guinea; 8 Engenderhealth, Conakry, Guinea

**Keywords:** Obstetric fistula, Factors associated, Repair, Failure, Incontinence, Guinea

## Abstract

**Background:**

The prevention and treatment of obstetric fistula still remains a concern and a challenge in low income countries.

The objective of this study was to estimate the overall proportions of failure of fistula closure and incontinence among women undergoing repair for obstetric fistula in Guinea and identify its associated factors.

**Methods:**

This was a retrospective cohort study using data extracted from medical records of fistula repairs between 1 January 2012 and 30 September 2013. The outcome was the failure of fistula closure and incontinence at hospital discharge evaluated by a dye test. A sub-sample of women with vesicovaginal fistula was used to identify the factors associated with these outcomes.

**Results:**

Overall, 109 women out of 754 (14.5 %; 95 % CI:11.9–17.0) unsuccessful repaired fistula at discharge and 132 (17.5 %; 95 % CI:14.8–20.2) were not continent.

Failure of fistula closure was associated with vaginal delivery (AOR: 1.9; 95 % CI: 1.0–3.6), partially (AOR: 2.0; 95 % CI: 1.1–5.6) or totally damaged urethra (AOR: 5.9; 95 % CI: 2.9–12.3) and surgical repair at Jean Paul II Hospital (AOR: 2.5; 95 % CI: 1.2–4.9).

Women who had a partially damaged urethra (AOR: 2.5; 95 % CI: 1.5–4.4) or a totally damaged urethra (AOR: 6.3; 95 % CI: 3.0–13.0) were more likely to experience post-repair urinary incontinence than women who had their urethra intact.

**Conclusion:**

At programmatic level in Guinea, caution should be paid to the repair of women who present with a damaged urethra and those who delivered vaginally as they carry greater risks of experiencing a failure of fistula closure and incontinence.

## Plain English summary

### Why was this study conducted?

This study was conducted to estimate the proportion of women who still leak urine after surgery for obstetric fistula and identify why the surgery was not successful in these women.

### How was the study conducted?

We evaluated three hospitals in Guinea where surgery for OF is conducted. We collected data from the medical records of women who got surgery for obstetric fistula between 1 January 2012 and 30 September 2013. For each woman, we determined whether or not she was still leaking continuously (unsuccessful surgery) or had some residual leakage (the hole was closed but still some leakage remains) after surgery and at the time the woman left the hospital.

Only women who had vesicovaginal or both vesicovaginal and recto-vaginal fistulas were considered for the identification of factors that explain why a woman stop leaking or not after surgery.

### What was found in this study?

The fistula was not closed in 109 women out of 754 (14.5 %) at the time they left the hospital. Overall 132 women (17.5 %) were still leaking urine either continuously or from time to time.

Women who delivered vaginally were two times as likely to continue leaking after surgery compared to women who delivered by C-section. Those with a partially damaged urethra or totally damaged urethra were respectively two times and six times as likely to continue leaking after surgery compared to women with a urethra not damaged.

### What have we learned?

During routine hospital repair of obstetric fistula in Guinea, surgeons should pay more attention to women who present with a damaged urethra and those who report having delivered vaginally because these kind of women are more difficult to treat than others.

## Background

Obstetric fistula is an abnormal opening between a woman’s vagina and bladder (vesicovaginal fistula, VVF), vagina and rectum (rectovaginal fistula, RVF) or vagina and both bladder and rectum (VVF + RVF) [1]. It is a serious morbidity that primarily follows obstructed labour and results in continuous and uncontrolled leakage of urine [1].

Despite international and national efforts, the prevention and treatment of obstetric fistula (OF) still remains a concern and a challenge in low income countries where access to emergency obstetric care and skilled birth attendant are insufficient [2, 3].

In sub-Saharan Africa, the lifetime prevalence of OF was estimated to be 1.60 per 1000 women of reproductive age (95 % CI 1.16, 2.10) [4] to 3.0 cases per 1000 (95 % CI: 1.3–5.5) [5].

Because of the smell of urine that results, women suffering of fistula are often abandoned by their spouses and relatives, keeping victims in poverty, isolation and depression [6, 7]. These women are also frequently exposed to medical complications such as infection, pain, sexual dysfunction and secondary infertility [8, 9].

The diagnosis of OF is done clinically through a pelvic exam verified by a dye test or cystoscopy and the treatment is mainly surgical through transvaginal or transabdominal techniques [10]. Surgical closure rates are reported to be as high as 90 % but vary from one repair hospital to another [2, 11, 12]. Rates also vary according to different characteristics including the denominator used (first time repair versus all repairs), repair technique, expertise of the surgeon, fistula characteristics and post-operative nursing care [13, 14]. In addition, authors might report the repair outcomes using different definitions [14, 15]. While some present the rates of fistula closure [16, 17], others clearly distinguish fistula closure and continence following repair surgery [2, 8]. As a result, comparisons of outcomes and performances from different contexts become difficult. To date there is no consensus on the classification of fistula and the definition of the outcomes of repair [13, 14].

Factors influencing the outcome of repair have been studied in different contexts. For instance, studies conducted by Barone et al. [17] with 1274 women in five countries in sub-Saharan Africa (including Guinea) and Asia, by Kayondo et al. [8] with 77 women at the referral regional hospital of Mbarara, Uganda, and by Nardos et al. [18] with 1045 fistula repairs conducted at the Addis Ababa Hamlin Fistula Hospital, showed that poor repair outcomes were significantly associated with complete urethral destruction, severe vaginal scarring, small bladders, and previous repairs.

While becoming continent after repair represents a rebirth for women suffering from fistula, failure of fistula closure can lead to further depression and isolation [19, 20]. In addition, repeat surgery for a fistula that has not been closed represents an additional social and economic burden for the woman and fistula care programmes as well as reduced likelihood of successful closure with subsequent repair attempts [21].

In Guinea, maternal health indicators are among the worst in Africa [22] and obstetric fistula is still prevalent [23]. From 2006 to 2013, more than 3000 repairs of female genital fistula (mostly OF) were supported by Engenderhealth in Guinea [24]. It is therefore important to document the country’s past experience in the management of fistula, especially in the most recent years, by identifying the factors that lead to unsuccessful closure or incontinence following successful fistula closure. This could inform and improve the implementation and performance of fistula care programmes, and contribute to improving the quality of services at repair hospitals. Even though Barone et al. [17] used data from Guinea in their study, the sample was not sufficient to allow country specific analysis, calling for additional research to fill the existing knowledge gap.

Therefore, the objective of this study was to estimate the overall proportions of failures of fistula closure and incontinence (following successful closure) at hospital discharge, and analyse their predictors in women who underwent repair for obstetric fistula in 2012 and 2013 in Guinea.

## Methods

### Study setting

Guinea is a coastal West African country with an estimated population of 10.5 million people, most of whom live in rural areas (65 %) and in poverty [25]. Guinea has an estimated lifetime prevalence of obstetric fistula of 0.6 % among women aged 15–49 with regional variations ranging from 0.2 to 1.2 %, although this figure is likely underestimated [23]. The country is characterised by a low national modern contraceptive prevalence (6 %), concurrent high fertility rate of 5.1 children per woman, and a high maternal mortality (724 deaths per 100,000 live births) [22, 26]. Over the period 2007–2012, the majority of births (54.7 %) were assisted by untrained individuals, and occurred at home (58.8 %) [23]. In 2013, Fistula repair services were available in five sites across the country, funded by international organizations such as UNFPA (Kankan Regional Hospital), WAHA International (Ignace Deen National Hospital) and EngenderHealth (Jean Paul II Hospital in Conakry, Labé Regional Hospital and Kissidougou Prefectural Hospital).

### Management of fistula at the repair hospitals

At EngenderHealth supported repair hospitals, fistula surgery was free and included assessment and surgical repair by a surgeon, and counselling about physical rehabilitation, family planning methods, and restrictions on sexual intercourse and heavy labour during the healing period and up to three months post-repair. Clinical procedures before, during and after surgery were harmonized in the three repair hospitals and included a pre-operative medical check-up, systematic use of antibiotics before and during (overall five days), and catheterization immediately after surgery for up to 21 days. Repairs were performed by a national team (sometimes assisted with surgeons from the GFMER) and local surgeons who were being trained. HIV infection or nutritional status were assessed but not captured in the medical records. Transportation costs to and from repair hospital were reimbursed and women were provided accommodation during the hospital stay. For each woman, surgical outcomes included failure of fistula closure (yes/no) and fistula incontinence (yes/no) were assessed immediately after surgery and at hospital discharge for all women. Data on the management of fistula surgeries at the other repair hospitals were not collected as part of this study.

### Study design

This was a retrospective cohort study examining the predictors of the failure of closure and urinary incontinence at discharge following the repair of obstetric fistula from 2012 to 2013 in three EngenderHealth supported repair hospitals in Guinea.

### Study population

The study population initially included all women who underwent repair for female genital fistula in the three hospitals between January 1st, 2012 and October 31, 2013. Women with the result of the dye test at discharge missing in medical records, non-obstetric fistula and duplicates i.e. previous repairs at a different study hospital were excluded. Only women with a vesicovaginal fistula were considered in the analysis of the predictors of repair failure.

### Data and key indicators

Study data were abstracted from patients’ files kept at each fistula care repair hospital and covered 22 months. The socio-demographic characteristics of fistula patients included age at presentation (in years), residence (rural or urban), marital status (married/union or other -single, divorced or widow-), woman’s occupation (housewife or worker) and level of education (none versus primary to higher). Gynecologic and clinical characteristics included parity, duration of labour (in days), mode of delivery (vaginal or caesarean section (CS)), neonatal outcome (alive or stillborn), and post-operative complications (fistula related or not). Fistula characteristics included type of fistula (vesicovaginal, rectovaginal, combined), vaginal scarring (yes or no), status of the urethra (intact, partially damaged or totally damaged), number of previous repairs and year of surgery. Treatment outcome was classified as follow: fistula closed (yes or no) and fistula continent (yes or no). Fistula was considered not closed when a woman had a positive dye test indicating continuous leakage of urine, as assessed by the surgeon, prior to hospital discharge. Women were considered to be incontinent at discharge when the result of the dye test was negative (fistula closed), but the woman reported a leakage of urine.

### Data analysis

Patients’ data were double entered by two independent data entry clerks into EpiData software (EpiData Association, Odense, Denmark), cleaned and exported for analysis in the STATA 13 software (STATA Corporation, College Station, TX, USA). We used the whole sample to summarise sociodemographic, clinical and fistula characteristics, and the outcome of repair with frequencies (%) and mean (with standard deviation). Pearson’s Chi square test (*χ* ^2^) was used to compare study outcomes across categorical variables and the Student *t*-test to compare the means of study outcomes across age, parity, duration of labour and previous repair. We considered the subsample of women with vesicovaginal fistula in the bivariable and multivariate analyses. Logistic regression models derived the unadjusted and adjusted odds ratios considering both the failure of fistula closure and fistula incontinence. All study variables with a *p*-value < 0.20 in the bivariable analysis were considered for inclusion in the logistic regression model. The significance level for the logistic regression model was set at 5 % with a 95 % confidence interval. The goodness of fit of the final model was tested by the Hosmer and Lemeshow test.

## Results

### Sociodemographic, clinical and fistula characteristics

**Fig. 1:**
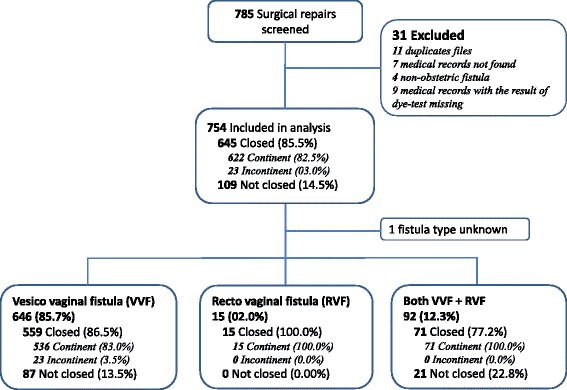
Patient flow and surgical repair outcomes by type of vaginal fistula at hospital discharge in three fistula repair hospitals supported by Engnderhealth in Guinea, 2012 to 2013

Overall 785 medical records of women who underwent surgical repair for female genital fistula at the three repair hospitals were screened of which 754 records were included in the analyses (Fig. 1). The demographic, gynecologic and clinical characteristics of these women are presented in Table 1. The mean age at presentation was 35.2 ± 12.7 years. The majority of women were married (*n* = 523, 69.4 %) with no formal education (691, 91.6 %) and lived in rural areas (677, 89.8 %). Most women delivered vaginally (*n* = 489, 64.9 %) after a mean duration of labor of 3.2 (±1.6) days. A total of 687 women (91.1 %) had stillbirths for the referent pregnancy. In terms of fistula characteristics, 419 women (55.6 %) had no previous attempt of repair and 389 women (51.6 %) had intact urethra. Few women (26 women; 3.6 %) developed postoperative complications including fistula related complications (15 women; 2.1 %).

**Table 1:** Demographic and clinical characteristics of women who underwent surgical repair of obstetric fistula in three Engenderhealth supported hospitals in Guinea, 2012 to 2013 (*N* = 754)

Variables	Number	Percentage
Total	754	100
Site		
Jean-Paul 2 Hospital	239	31.7
Labe regional Hospital	199	26.4
Kissidougou Distric Hospital	316	41.9
Mean age at presentation to repair centre (SD), years	35.2(12.7)	
Residence		
Rural	677	89.8
Urban	75	09.9
Unknown	2	00.3
Marital status		
Married/Union	523	69.4
Other (single, separated, widow)	208	27.6
Unknown	23	03.0
Occupation		
Housewife	711	94.3
Worker	25	03.3
Unknown	18	02.4
Education		
None	691	91.6
Primary and more	55	07.3
Unknown	8	01.1
Mean parity (SD), deliveries	3.6(2.6)	
Mean duration of labour (SD), days	3.2(1.6)	
Mode of delivery		
Vaginal	489	64.9
Caesarean section	237	31.4
Unknown	28	03.7
Neonatal outcome at delivery		
Alive	40	05.3
Stillborn	687	91.1
Unknown	27	03.6
Vaginal scarring		
Yes	373	49.2
No	371	49.5
Unknown	10	01.3
Status of the urethra		
Intact	389	51.6
Partially damaged	264	35.0
Totally damaged	88	11.7
Unknown	13	01.7
Previous surgical repair		
None	419	55.6
One	190	25.2
Two or more	137	18.2
Unknown	8	01.0
Route of repair		
Vaginal	732	97.1
Abdominal	14	01.8
Combined	6	00.8
Unknown	2	00.3
Year or repair		
2012	489	64.8
2013	265	35.2
Post-operative complications^a^		
None	728	96.5
Fistula related	15	02.0
Others	11	01.5

*SD* standard deviation, *VVF* vesicovaginal fistula, *RVF* rectovaginal fistula

^a^Fistula related complication: Haemorrhage (7), Wound infection (4), Urine retention (3), and Urethral narrowing (1). Other complications: Malaria (7), Hypertensive disorders (2), Diarrhoe (1), and Zona (1)

### Study treatment outcomes

Figure 1 shows the study flow and repair outcomes at the time of hospital discharge by type of fistula. Overall, 109 women out of 754 (14.5 %; 95 % CI:11.9–17.0) had unsuccessful repairs at discharge and 23 additional women (3.0 %; 95 % CI:1.8–4.2) had successful repairs but remained incontinent meaning that overall 132 (17.5 %; 95 % CI:14.8–20.2) were not continent. Of the 646 women who had VVF (86.5 % of the study sample), 87 (13.5 %; 95 % CI:10.9–16.1) had unsuccessful repairs and 23 (3.5 %; 95 % CI:2.1–4.9) remained incontinent. The 15 women who had an RVF (2.1 % of the sample) had successful repairs and were continent of stools. As for the 92 women who had both VVF and RVF (12.3 % of the sample), 21 (22.8 %; 95 % CI:14.2–31.4) had unsuccessful repairs.

### Context of repair

The context of repair at the three hospitals where women were managed is presented in Table 2. Between 2012 and 2013, the three sites benefited from trainings in fistula repair and care, infection prevention and emergency obstetric care. The CS rate was significantly higher in Kissidougou hospital as compared to the other two hospitals. Overall, the systematic post-operative antibiotic treatment (up to 5 days) was high at the sites (from 96 to 100 %) with no statistically significant difference across repair hospitals. However, 51.1 % (45/88) of the women with totally damaged urethra and 79.4 % (73/92) of the combined (VVF + RVF) fistulas were repaired at the Conakry Jean Paul II Hospital (*p* < 0.001).

**Table 2:** Selected characteristics of three Engenderhealth supported fistula repair hospitals in 2013 in Guinea

Variables	Hospitals		
Total	Jean-Paul II (Conakry)	Labe	Kissidougou
Fistula repair and care training			
Number of Fistula Surgeons	03	03	03
Number of Nurses and Midwives	12	13	07
Number of Anesthetists	02	03	03
Infection prevention training (number of attendees)	10	18	16
Obstetric care training (number of attendees)	20	17	17
Workload			
Annual number of deliveries	2024	2795	2426
Annual number of Caesarean sections (%)	342 (16.9 %)	762 (27.3 %)	791 (32.6 %)
Existence of fistula ward			
Yes	Yes	Yes	Yes
Number of beds dedicated to fistula	32	20	14
Infection prevention measures			
Sterilisation	Yes	Yes	Yes
Type of sterilisation	Autoclave	Autoclave	Autoclave
Post-operative antibiotic treatment			
Proportion of use^a^	95.7	100	98.7
Post-operative fistula related complications (number and %)^a^	7 (2.94 %)	3 (1.51 %)	5 (1.76 %)
Status of the urethra			
Intact	130 (56.5 %)	89 (44.7 %)	170 (54.5 %)
Partially damaged	55 (23.9 %)	81 (40.7 %)	128 (41.0 %)
Totally damaged	45 (19.6 %)	29 (14.6 %)	29 (4.5 %)
Type of fistula			
Vesicovaginal fistula (VVF)	159 (66.5 %)	182 (91.5 %)	305 (96.5 %)
Rectovaginal fistula (RVF)	6 (2.5 %)	5 (2.5 %)	4 (1.3 %)
Both VVF and RVF	73 (31.0 %)	12 (6.0 %)	7 (2.2 %)

^a^from the dataset

### Factors associated with repair failure

#### Failure of fistula closure

In bivariable analysis, we found that mean age at presentation, mode of delivery, vaginal scarring, status of the urethra and repair hospital were statistically significantly associated with the failure of fistula closure (Table 3). However, in multivariate analysis, only mode of delivery, status of the urethra and repair hospital were independently associated with the failure of fistula closure. The odds of experiencing failure of fistula closure were higher among women who delivered vaginally as compared to women who delivered by CS (AOR: 1.9; 95 % CI: 1.0–3.6). Women who had their urethra partially (AOR: 2.0; 95 % CI: 1.1–5.6) or totally damaged (AOR: 5.9; 95 % CI: 2.9–12.3) were more likely to have a fistula not closed as compared to women who had an intact urethra. Women who were repaired at Jean Paul II Hospital were more likely to have a fistula not closed at discharge than women repaired at Kissidougou hospital (AOR: 2.5; 95 % CI: 1.2–4.9).

**Table 3:** Logistic regression of the failure of fistula closure and incontinence among women with vesicovaginal fistula repaired in three Engenderhealth supported repair hospitals from 2012 to 2013 in Guinea (*N* = 646)

Variables	Failure of fistula closure (Yes)			Fistula incontinence (Yes)		
	N (%)	Unadjusted OR [95 % CI]	Adjusted^a,b^ OR [95 % CI]	N (%)	Unadjusted OR [95 % CI]	Adjusted^a,c^ OR [95 % CI]
Mean age at presentation (SD), years	38.9 (14.1)	1.02 (1.00–1.04)	1.02 (1.00–1.04)	38.2 (13.6)	1.02 (1.00–1.03)	1.01 (0.99–1.03)
Residence						
Rural	81 (13.4)	1	-	101 (16.7)	1	-
Urban	6 (14.0)	1.0 (0.4–2.5)		9 (20.9)	1.3 (0.6–2.8)	
Marital status						
Married/Union	55 (11.9)	1	1	70 (15.2)	1	1
Other (single, separated, widow)	29 (16.9)	1.5 (0.9–2.4)	1.1 (0.6–1.9)	36 (21.0)	1.5 (0.9–2.3)	1.2 (0.7–2.1)
Occupation						
Housewife	82 (13.4)	1	-	104 (17.0)	1	-
Worker	3 (15.8)	1.2 (0.3–4.2)		4 (21.0)	1.3 (0.4–4.0)	
Education						
None	79 (13.3)	1.0 (0.4–2.5)	-	102 (17.2)	1.4 (0.6–3.4)	-
Primary and more	6 (12.8)	1		6 (12.8)	1	
Mean parity (SD), deliveries	3.6 (2.8)	0.97 (0.89–1.06)	-	3.5 (2.8)	0.95 (0.87–1.03)	1.00 (0.90–1.11)
Mean duration of labor (SD), years	3.4 (1.6)	1.10 (0.95–1.28)	-	3.4 (1.5)	1.08 (0.95–1.24)	-
Mode of delivery						
Vaginal	65 (15.7)	2.3 (1.3–4.0)	1.9 (1.0–3.6)	80 (19.3)	1.9 (1.1–3.0)	1.5 (0.9–2.6)
Caesarean section	16 (7.6)	1	1	24 (11.4)	1	1
Neonatal outcome at delivery						
Alive	2 (6.9)	1	-	2 (6.9)	1	1
Stillborn	82 (13.6)	2.1 (0.5–9.1)		105 (17.5)	2.9 (0.7–12.2)	2.0 (0.4–9.1)
Vaginal scarring						
Yes	56 (16.8)	1.9 (1.1–2.9)	1.2 (0.7–2.0)	73 (21.9)	2.0 (1.3–3.1)	1.3 (0.7–2.1)
No	31 (10.1)	1	1	37 (12.1)	1	1
Status of the urethra						
Intact	26 (7.6)	1	1	32(9.4)	1	1
Partially damaged	34 (14.5)	2.1 (1.2–3.5)	2.0 (1.1–5.6)	49 (20.9)	2.6 (1.6–4.1)	2.5 (1.5–4.4)
Totally damaged	27 (42.9)	9.1 (4.8–17.3)	5.9 (2.9–12.3)	29 (46.0)	8.3 (4.5–15.3)	6.3 (3.0–13.0)
Previous surgical repair	0.83 (0.99)	1.11 (0.90–1.38)	-	0.92 (1.13)	1.23 (1.02–1.48)	1.04 (0.83–1.31)
Year or repair						
2012	62 (15.2)	1.5 (0.9–2.5)	1.0 (0.6–1.8)	78 (19.1)	1.5 (1.0–2.4)	1.0 (0.6–1.6)
2013	25 (10.5)	1	1	32 (13.4)	1	1
Fistula related post-op complications						
No	84 (13.2)	1	1	107 (16.8)	1	-
Yes	3 (30.0)	2.8 (0.7–11.1)	2.9 (0.5–15.9)	3 (30.0)	2.8 (0.8–9.8)	
Repair site						
Jean-Paul 2 Hospital	33 (20.8)	2.7 (1.5–4.7)	2.5 (1.2–4.9)	36 (22.6)	1.6 (1.0–2.6)	1.6 (0.8–3.1)
Labe regional Hospital	27 (14.8)	1.8 (1.0–3.2)	1.6 (0.9–3.0)	27 (14.8)	1.0 (0.6–1.6)	0.8 (0.5–1.4)
Kissidougou District Hospital	27 (8.9)	1	1	47 (15.4)	1	1

*SD* standard deviation, *OR* odds ratios, *CI* confidence intervals, *N* number

^a^Multivariate analysis adjusting for all confounding factors with *p*-value < 0.20 in bivariable analysis

^b^Hosmer-Lemeshow test for goodness-of-fit: chi2 (8 d.f.) = 8.9; *p* = 0.369

^c^Hosmer-Lemeshow test for goodness-of-fit: chi2 (8 d.f.) = 8.2; *p* = 0.411

#### Incontinence following successful closure

Bivariable analysis showed that age at presentation, mode of delivery, vaginal scarring, status of the urethra, previous surgical repair and repair hospital were statistically significantly associated with fistula incontinence. However, in multivariate analysis, only status of the urethra remained statistically significantly associated with fistula incontinence. Women who had a partially damaged urethra (AOR: 2.5; 95 % CI: 1.5–4.4) or a totally damaged urethra (AOR: 6.3; 95 % CI: 3.0–13.0) were more likely to experience post-repair urinary incontinence than women who had their urethra intact.

## Discussion

This study provides an overview of the factors associated with the failure of obstetric fistula repair at hospital discharge in Guinea, adding to the existing evidence on the subject.

The overall proportion of the failure of fistula closure observed in this study (14.5 %) is lower than what has been reported in different contexts in Africa [8, 17, 18]. These findings might be explained by differences in the level of complexity of fistula cases managed and/or characteristics of the repair hospitals (experience and skills of the staff, pre and post-surgical clinical procedures, infection prevention measures in place) [14, 15]. Barone et al. [17] who conducted their study in five countries including Guinea did not provide such information and did not stratify their results by country.

We found that failure of fistula closure varied significantly across repair hospitals. Because the three repair hospitals were part of the same project, with the same trainings, clinical procedures, equipment and trainer surgeons, the differences observed cannot be attributed to hospital characteristics unless the difference relates to differing skill levels among surgeons or to the characteristics of the fistulas repaired for which we could not adjust for in this analysis [14, 15]. It is also possible that differences in the uptake or implementation of trainings and interventions differed by repair hospital, which may have influenced the results. However, our data shows that Jean Paul II hospital in Conakry received more fistula with ‘worse’ characteristics than the two other hospitals, suggesting greater damage. The reasons might be because: 1) The hospital is situated in the Capital city where the national fistula trainers live, 2) It was the referral centre for fistula care during the Engenderhealth’s Fistula Care Project.

After adjusting for confounding factors that were measured, we observed that failure of fistula closure was associated with mode of delivery, status of the urethra and repair hospital. However, fistula incontinence was only statistically significantly associated with the status of the urethra. Because the duration of labour was high for the women of this study (3 days or more), it is likely that the fistula already begins to develop before women are referred for a CS. Therefore, for women who benefit a CS, the extension of the fistula to involve the urethra is limited as compared to those who still deliver vaginally.

Previous studies have reported that urethral involvement might affect sphincter mechanisms and bladder size and therefore cannot be easily addressed surgically, leading to more repair failure [8, 17, 18, 27]. Vaginal scarring has been reported to be predictive of the failure of fistula closure [8, 17, 18]. However, the associations found in the bivariable analyses in our study were not sustained in multivariate analyses.

We found that fistula related postoperative complications were not associated with both failure of fistula closure and fistula incontinence in both bivariable and multivariate analyses. Complications such as urine retention, wound infection and haemorrhage are likely to be linked to quality of services including quality of surgery, implementation of infection prevention measures or postoperative care and duration of catheterization [28]. Overall the occurrence of fistula related postoperative complications was low and the difference between repair hospitals was not statistically significant. This might be because antibiotics were systematically used before, during and after fistula surgery in Engenderhealth supported hospitals. Monitoring the quality of surgery and improving and sustaining quality postoperative care are needed to maintain surgeon’s performance. Even though EngenderHealth had staff dedicated to its programmes with regular onsite trainings and monitoring visits, including on infection prevention measures, there might still be room for improvement.

In our study, most patient characteristics did not independently predict the failure of fistula closure nor fistula incontinence. This has previously been reported from different contexts in the literature [8, 11, 17, 18, 29]. Finally, we did not include fistula size as a variable because its assessment was unreliable in our dataset. However, in the existing literature, no relationship between fistula size and failure of fistula closure or incontinence is reported [15, 29–31].

Our study had some limitations: 1) as an observational study, it is possible that all confounding factors were not examined or controlled for; 2) because some variables such as demographic variables were self-reported, there was a possibility of inaccuracy and reporting bias; 3) there were some missing values for variables examined in the bivariable and multivariate analyses; 4) it was not clear how some fistula characteristics were recorded and there were no standard definition of complications; 5) the experience and skills of providers were not assessed and; 6) we could not account for the amount of time between surgery and discharge across repair hospitals.

However, this is one of the few studies to examine the predictors of fistula repair failure in Guinea and West Africa.

## Conclusions

This study shows that in Guinea, status of the urethra was an independent predictor of failure of fistula closure and incontinence in women at discharge while mode of delivery was also a predictor of failure of fistula closure. Therefore, caution should be made when women present for repair with such characteristics. In addition, exploring others approaches such as the use of mixed methods in realist evaluation designs might provide additional insights on other factors/predictors not explored in our study [32].

## Abbreviations

ANC: Antenatal care
CI: Confidence intervals
IRB: Institutional Review Board
ITM: Institute of Tropical Medicine
OF: Obstetric fistula
RVF: Rectovaginal fistula
UNFPA: United Nations Population Fund
USA: United States of America
USAID: United States Agency for International Development
VVF: Vesicovaginal fistula
